# Remote psychophysical testing of smell in patients with persistent olfactory dysfunction after COVID-19

**DOI:** 10.1038/s41598-023-41395-9

**Published:** 2023-08-28

**Authors:** Marcela Martončíková, Pavel Doležal, Kamila Fabianová, Miloslav Karhánek, Ján Gálik, Adam Raček, Alexandra Popovičová, Enikő Račeková

**Affiliations:** 1 Institute of Neurobiology, Biomedical Research Center, Slovak Academy of Sciences, Šoltésovej 4, 040 01 Košice, Slovakia; 2 Department of Otorhinolaryngology and Head and Neck Surgery, University Hospital-St. Michal’s Hospital, Slovak Medical University, Bratislava, Slovakia; 3 Laboratory of Bioinformatics, Biomedical Research Center, Slovak Academy of Sciences, Dúbravská Cesta 9, 845 05 Bratislava, Slovakia

**Keywords:** Diseases, Medical research

## Abstract

Olfactory dysfunction associated with coronavirus 2 (SARS-CoV-2) infection is in most cases transient, recovering spontaneously within a few days. However, in some patients it persists for a long time, affects their everyday life and endangers their health. Hence, we focused on patients with persistent loss of smell. The aim of this study was to evaluate olfactory dysfunction using a standardized test. Due to the pandemic, olfactory testing was performed online. Smell tests (Odorized Markers Test, OMT) were sent home to the patients. Together with the smell self-testing, participants reported and assessed several parameters (age, sex, subjective assessment of smell and taste, nasal patency, etc.) in an online questionnaire. Based on the questionnaire outcomes, the results were sent to the patients along with a list of participating otolaryngologists who provided them with professional care. From March to June 2021, 1025 patients requested smell testing, of these, 824 met the inclusion criteria of this study. The duration of the olfactory dysfunction at the time of testing ranged from 1 month to 1 year. Using the OMT, impaired smell ability—anosmia or hyposmia—was confirmed in 82.6% of participants. A total of 17.4% of participants were determined to be normosmic however, more than 50% of them complained of parosmia and/or phantosmia. Our study demonstrates the relevance of psychophysical smell testing and its suitability for remote use during the pandemic. This study also revealed several correlations between prolonged olfactory dysfunction and the monitored parameters.

## Introduction

The loss of sense of smell and/or taste was recognized shortly after the outbreak of the pandemic as one of the symptoms of coronavirus disease 2019 (COVID-19) caused by coronavirus 2 (SARS-CoV-2). The World Health Organization added "loss of taste or smell" to its list of the symptoms of COVID-19 at the beginning of May 2020. According to a meta-analysis, the worldwide prevalence of dysfunctions in smell and taste among COVID-19 patients was 49%^1–3^. The prevalence of chemosensory deficits among COVID-19 patients in Central Europe was 50–75%^3,4^.

Olfactory disorder induced by SARS-CoV-2 has specific features. Its onset is sudden and may not be accompanied by nasal congestion or rhinorrhea. Loss of sense of smell is one of the early symptoms of SARS-CoV-2 infection, but it is more predictive of a COVID-19 diagnosis than other early symptoms^5^. In some patients, the loss of smell and/or taste is the only symptom of COVID-19 infection^6,7^.

Great efforts have been made to identify the pathophysiology and underlying mechanism of olfactory dysfunction (OD). The question is why in some patients the loss of smell is transient, while in others, it persists for a long time. SARS-CoV-2 enters cells by binding its spike protein to the ACE-2 receptor on target cells^8^. ACE-2 together with the protease TMPRSS2 is expressed on sustentacular cells of the olfactory epithelium but not on sensory olfactory neurons^9,10^. Based on immunohistochemistry, it has been demonstrated that ACE-2 expression is 200–700 times higher in the sustentacular cells of the olfactory neuroepithelium relative to nasal respiratory or tracheal epithelial cells^11^. Although they are not sensory cells, sustentacular cells play an important role in peripheral processing of odor molecules^3^. In addition to physical support, these cells increase the sensitivity of olfactory receptor neurons by odorant clearing function^12^ and may supply them with glucose to cover energy demands concerning olfactory signal transduction^13^. Thus, virus-induced destruction of supporting cells may impair olfactory abilities, whereas olfactory receptor neurons do not have to be damaged^14^. Regeneration of supporting sustentacular cells occurs much faster than regeneration of olfactory receptor neurons^14,15^. Moreover, the time course of regeneration of sustentacular cells is consistent with regaining the sense of smell observed in most cases^3^. In addition, a genetic link to the biological mechanisms underlying COVID-19-related acute loss of smell or taste has been demonstrated. Polymorphisms in the UGT2A1/UGT2A2 locus, whose gene products are the enzymes involved in the elimination of the odorants that enter the nasal cavity and bind to olfactory receptors, are associated with COVID-19-loss of smell or taste^16^. The facts mentioned above could explain transient loss of smell caused by SARS-CoV-2 infection.

Anosmia could also be caused by a "cytokine storm". Immune cell infiltration into the olfactory epithelium has been shown as a consequence of SARS-CoV-2 infection^14^, and this infiltration appears to be accompanied by a substantial increase in proinflammatory cytokines^17^. Although there are no ACE-2 receptors on olfactory receptor neurons, they can be affected secondarily due to damage to sustentacular cells^18,19^. Massive infiltration of immune cells in the OE and lamina propria might lead to the destruction of the OE and to loss of noninfected olfactory sensory neurons^14,20^. Longer-lasting anosmia may result from affecting a larger area of olfactory epithelium or a more profound destruction of the epithelium including death of olfactory receptor neurons^3^. Persistent anosmia may also be related to the neurotropic potential of SARS-CoV-2. Olfactory sensory neurons represent direct pathway to the brain by anterograde axonal transport. Recently, a neuropilin-1 receptor (NRP1), capable of binding to the SARS-CoV-2 spike protein, has been found on olfactory sensory neurons as well as on their progenitor cells^21^. Viral entry mediated by NRP1 may facilitate direct injury of olfactory sensory neurons, loss of progenitor cells and axonal transport to the olfactory bulb and this mechanism may be responsible for delayed or absent olfactory recovery^22^. Moreover, biopsy-based analysis has revealed that T-cell-mediated inflammation persists in the olfactory epithelium long after SARS-CoV-2 infection and might cause persistent post-COVID-19 loss of smell^23^.

Women report olfactory and gustatory dysfunctions more frequently than men^24^, and its occurrence is more typical among younger people. Smell and/or taste deficits linked with COVID-19 are usually transient lasting up to 2 weeks^3,7^. However, in some patients (10–17%) olfactory and/or gustatory deficits persist for a long time^25–29^. During the pandemic, when hundreds of millions of people were infected with the SARS-CoV-2, this percentage means that there are a large number of people with OD. As in the rest of the world, in Slovakia, persistent OD affected a number of patients. Even if the olfactory disorder is not life-threatening, it can significantly worsen the quality of life and negatively affect the patient's health.

Self-report of chemosensory function is subjective and may not be sufficiently reliable. This applies especially to self-reported taste dysfunction^30^ but also applies to self-reported evaluation of smell^31^. Indeed, studies that highlighted the clinical importance of odor testing in COVID-19 early in the pandemic revealed significant differences between self-reports of olfaction and objective olfactory testing in COVID-19^32–34^. Unfortunately, psychophysical olfactory testing has not been established in clinical practice in Slovakia. Due to the increasing number of patients with persistent OD after COVID-19, there was the need to evaluate the olfactory disorder with standardized smell tests. For these reasons the project "Smell and COVID-19" was designed in which a network of otolaryngologists (ear, nose, and throat (ENT) doctors) also participated.

The aim of the project was to test the smell of post-COVID-19 patients with persistent OD with standardized smell tests and refer them with outcomes to the ENT doctors involved, as well as to raise awareness of the issue of olfactory loss. Due to the pandemic, olfactory testing could take place only remotely. For this purpose, a website was created where it was possible to register for self testing. A simple six item smell test for odor identification, the Odorized Markers Test (OMT), was sent to the addresses provided by enrolled participants. According to the results of the psychophysical smell test, anosmia, hyposmia and normosmia could be recognized in individual participants. Outcomes of the test were sent back to the participants with recommendations to visit participating ENT doctors.

This study was based on the analysis of data provided by participants in the olfactory self-test and in the online questionnaires. The questionnaire included questions about: age, sex, weight, the date of onset of COVID-19-associated OD, self-assessment of smell, taste and nasal patency, and occurrence of parosmia and phantosmia.

## Results

### Participant characteristics

Post-COVID-19 patients with persistent olfactory deficits (n = 824; median age 40 years; range 10–80 years); 598 (72.5%) women (median age 41.5 years; range 10–80 years) and 226 (27.4%) men (median age 35 years; range 13–75 years) completed the olfactory test and questionnaire in this study.

The average age of the tested subjects was 39.5 years and the median age was 40.0 years, while the average ages of the women and men were 40.6 and 36.6 years, respectively.

**Figure 1:**
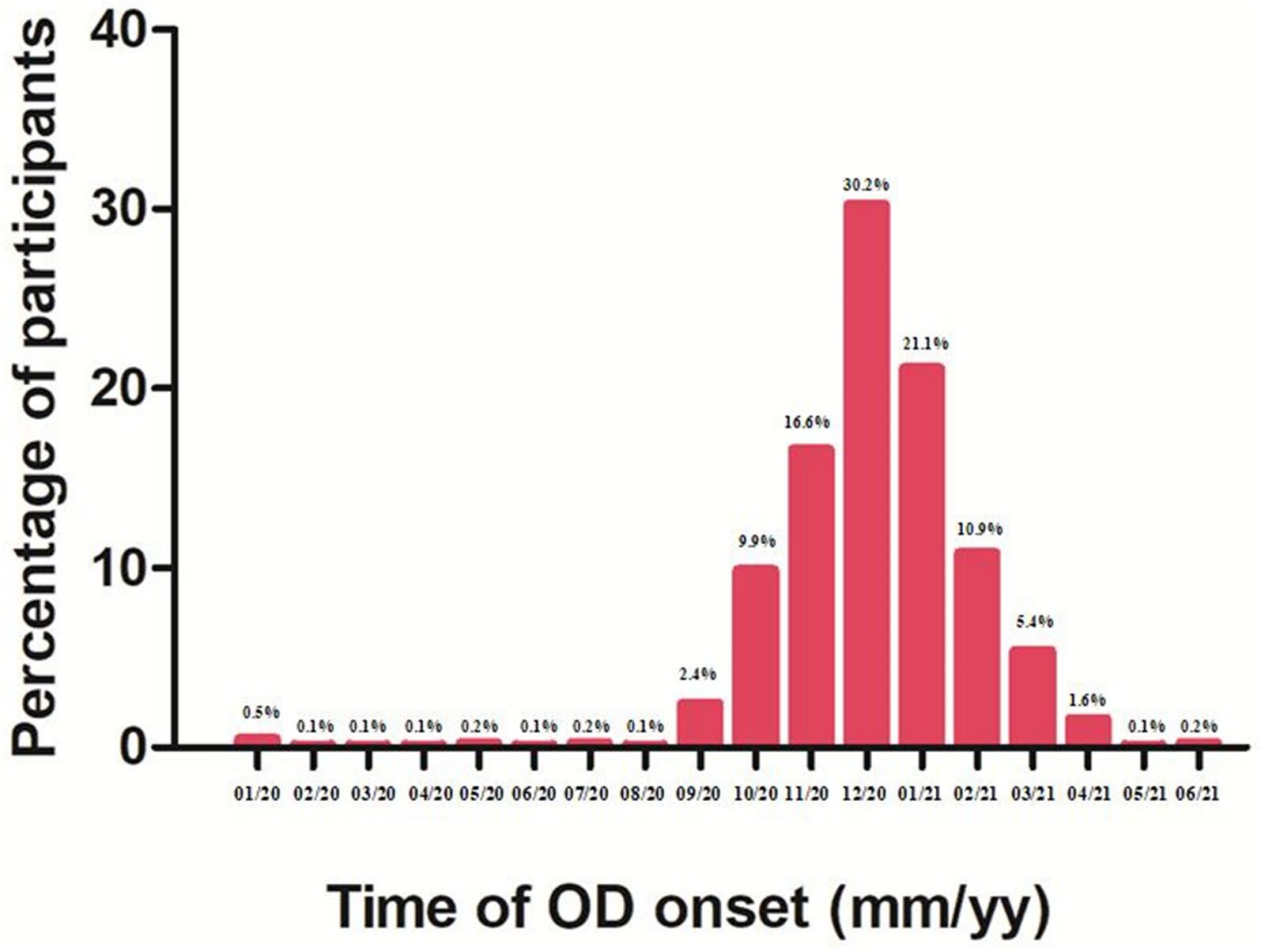
The percentage of participants according to the time of OD onset. Note the peak between November 2020 and February 2021.

The participants were asked to indicate the time (month and year) when they first noticed their OD associated with COVID-19 infection. The data provided by 820 participants (4 participants were excluded due to missing OD onset information) are summarized in Fig. 1 (Supplementary Table 1). According to these data, most participants lost their sense of smell between November 2020 and February 2021.

### Quantitative olfactory dysfunction: OMT-based classification of normosmia, hyposmia, and anosmia

**Figure 2:**
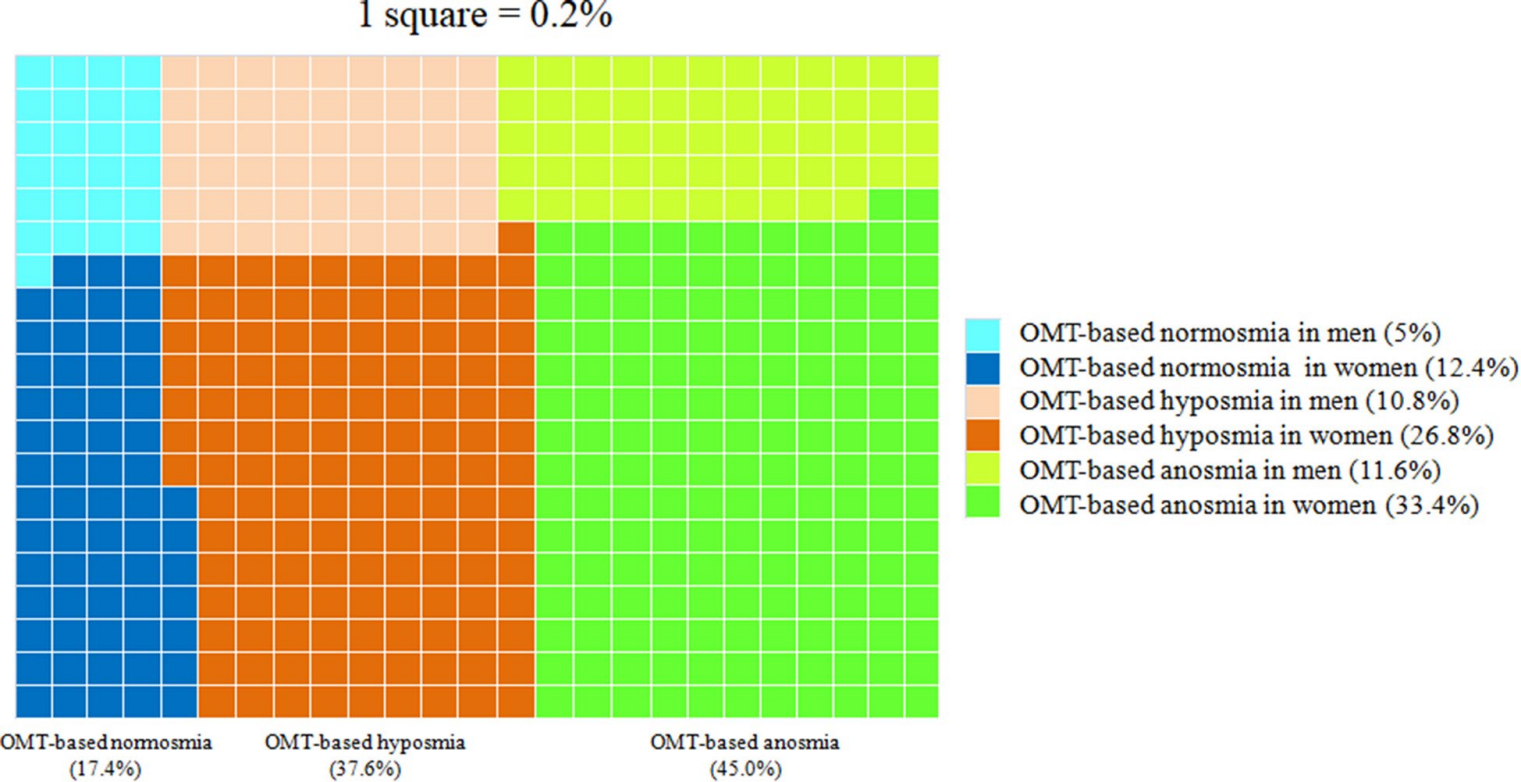
Percentage of OMT-based normosmia, hyposmia and anosmia. According to the OMT, 17.4% (women = 12.4%, men = 5%), 37.6% (women = 26.8%, men = 10.8%) and 45.0% (women = 33.4%, men = 11.6%) of participants were normosmic, hyposmic and anosmic, respectively.

To evaluate quantitative olfactory dysfunctions, participants self-administered six spice and fruit odor identification tests that can distinguish among normosmia and quantitative olfactory dysfunctions, hyposmia and anosmia (for more detailed information on the OMT, see the “Materials and methods”). Based on the OMT scores, we classified the 824 participants into OMT-based normosmia (n = 143, 17.4%, OMT score 9–12), OMT-based hyposmia (n = 310, 37.6%, OMT score 6–8), and OMT-based anosmia (n = 371, 45.0%, OMT score 0–5) (Fig. 2). This classification revealed that in Slovakia one fourth (26.8% = 221/824) and one third (33.4% = 275/824) of the participants with persistent olfactory dysfunction included in this study were women OMT-based hyposmia and OMT-based anosmia, respectively (Fig. 2).

**Figure 3:**
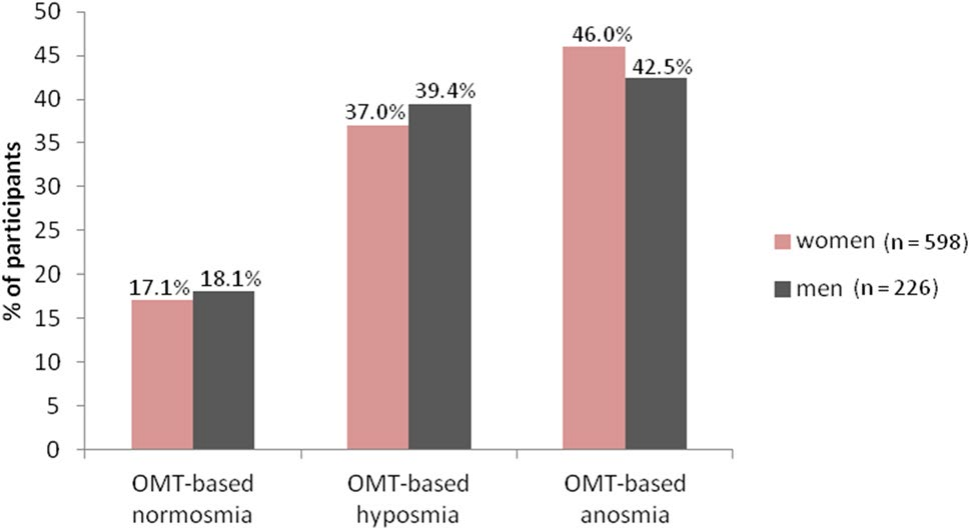
OMT outcomes according to sex.

Regarding sex, men had slightly higher incidences of OMT-based normosmia (18.1% = 41/226) and OMT-based hyposmia (39.4% = 89/226) than women (17.1% = 102/598 and 37.0% = 221/598, respectively), whereas women had a higher incidence (46.0% = 275/598) of OMT-based anosmia than men (42.5% = 96/226) (Fig. 3).

### Self-reported olfactory dysfunction vs. OMT score

Self-reports of smell using a numeric scale (1 = no perception and 10 = perfect perception) were compared with the OMT outcomes of participants to evaluate the reliability of the self-report. The correlation coefficient (r) between self-reports and OMT scores of participants was 0.536 (p < 0.05), i.e. the self-report did not correspond with the OMT score in all cases. The comparison showed that 33.6% of people with OMT-based normosmia (with OMT scores 9–12) substantially underestimated their olfaction (they rated their sense of smell on the numeric scale by a number less than 5). Conversely, 18.9% of people with OMT-based anosmia (with OMT scores less than 5) markedly overestimated their olfactory abilities (they evaluated their sense of smell on a numeric scale from 5 to 10).

### Olfactory dysfunction and its association with self-reported nasal patency

Nasal patency can be reduced by nasal congestion caused by local inflammation associated with rhinorrhea and mucosal edema during infection of the upper airways. This can reduce or stop airflow through the nasal cavity which prevents the transmission of odorants to the olfactory epithelium in the upper part of the nasal cavity and might result in conductive loss of smell, i.e. olfactory dysfunction. We analyzed whether olfactory dysfunction in post-COVID-19 patients was related to the subjectively perceived changes in nasal patency at the time of infection. Based on the self-evaluation of smell on numeric scale from 1 (no perception) to 10 (perfect perception), 89.0% (733/824) of the participants rated their smell ability during COVID-19 by a value < 10. From this group of participants, 20.1% (147/733) rated their nasal patency on numeric scale during COVID-19 by the highest value (10 = free breathing through the nose). This suggests that their olfactory dysfunction was not related to their self-reported nasal congestion. A total of 79.9% (586/733) of the participants rated their nasal patency with a value < 10, suggesting that their olfactory dysfunction may also result from reduced nasal patency.

**Figure 4:**
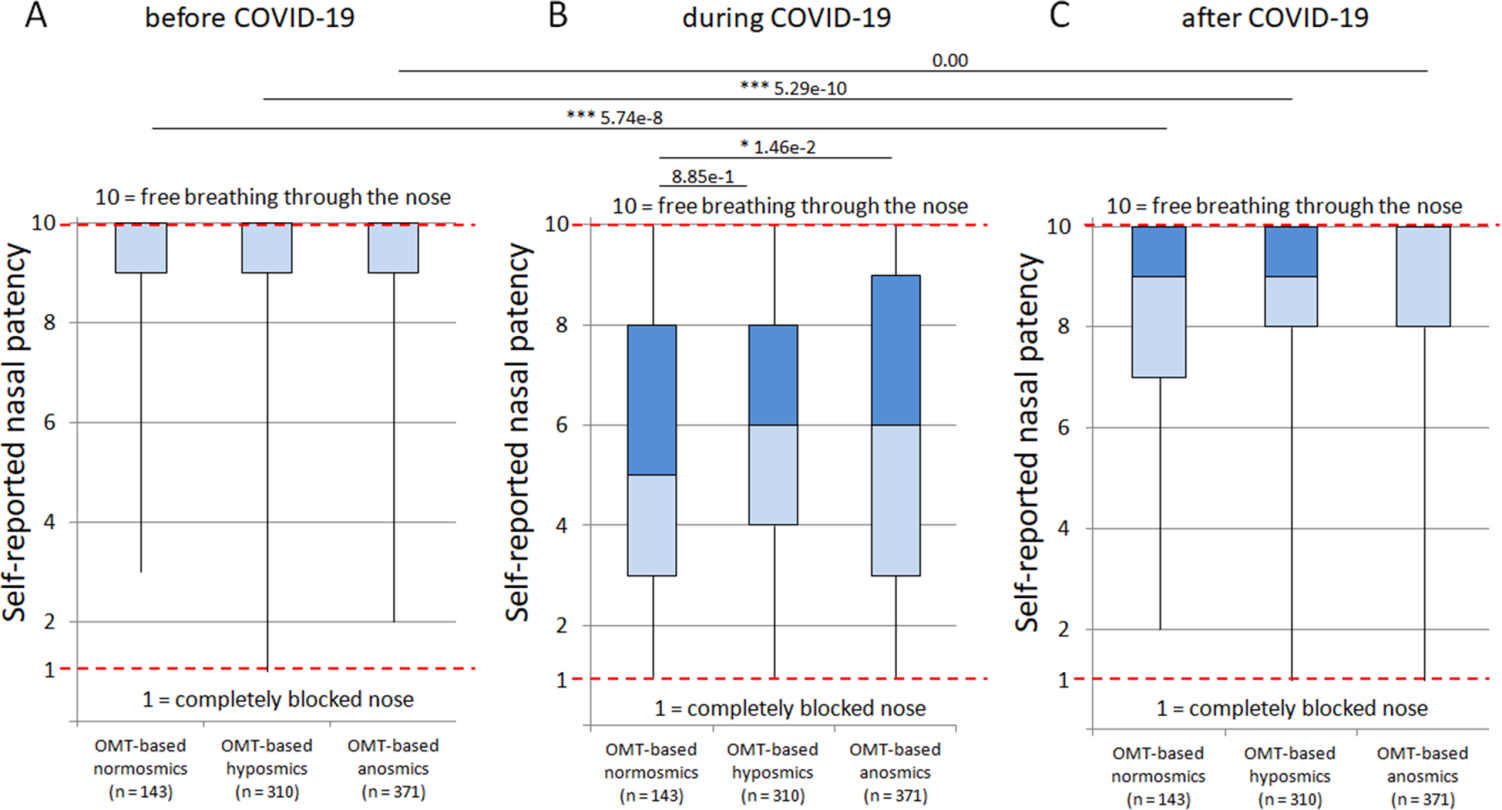
Self-reported nasal patency in OMT-based groups with normosmia, hyposmia and anosmia, before (**A**), during (**B**), and after (**C**) COVID-19. Significant decreases in self-reported nasal patency during COVID-19 occurred in all OMT-based groups compared to those before and after COVID-19 (P < 0.001). During COVID-19, self-reported nasal patency worsened in OMT-based normosmic participants more than in OMT-based anosmic participants (**B**). After COVID-19, self-reported nasal patency did not recover to the levels before COVID-19 in OMT-based normosmic participants and OMT-based hyposmic participants but did in OMT-based anosmic participants (**C**). A sex comparison of the subjective rating of nasal patency in OMT-based anosmic and OMT-based hyposmic participants showed some differences between men and women, but they were not statistically significant according to the Tukey‒Kramer test (Supplementary Fig. 1).

Figure 4 shows self-reported nasal patency on a numeric scale (from 1 to 10; 1 means completely blocked nose and 10 means free breathing through the nose) before (Fig. 4A), during (Fig. 4B) and after COVID-19 (Fig. 4C) in individual OMT-based groups with normosmia, hyposmia and anosmia. The Tukey‒Kramer test revealed significant decreases (P < 0.001) in self-reported nasal patency during COVID-19 in all three OMT-based groups compared to those before and after COVID-19 (Fig. 4). During infection, OMT-based anosmic participants rated their nasal patency with higher numeric scores than OMT-based normosmic participants, indicating that they had better nasal patency than OMT-based normosmic participants (Fig. 4B). After COVID-19, anosmic participants rated their nasal patency with higher numeric scores than hyposmic and normosmic participants (Fig. 4C). Self-reported nasal patency did not recover to the levels before COVID-19 in OMT-based normosmic or hyposmic participants but did in OMT-based anosmic participants after COVID-19 (Fig. 4A,C). Based on Spearman correlation analysis, negative correlations were found between the OMT score and self-reported nasal patency (r = − 0.078 and r = − 0.111) of participants during and after COVID-19, respectively. These results suggest that OD caused by SARS-CoV-2 infection does not depend on reduced nasal patency.

### Severity-dependent persistence of olfactory dysfunction

Due to the large differences in the duration of OD, which ranged from 1 to 14 months after COVID-19, we divided the participants into three groups for further analysis: 1–2 months; 3–5 months; and 6 or more months of OD duration.

**Figure 5:**
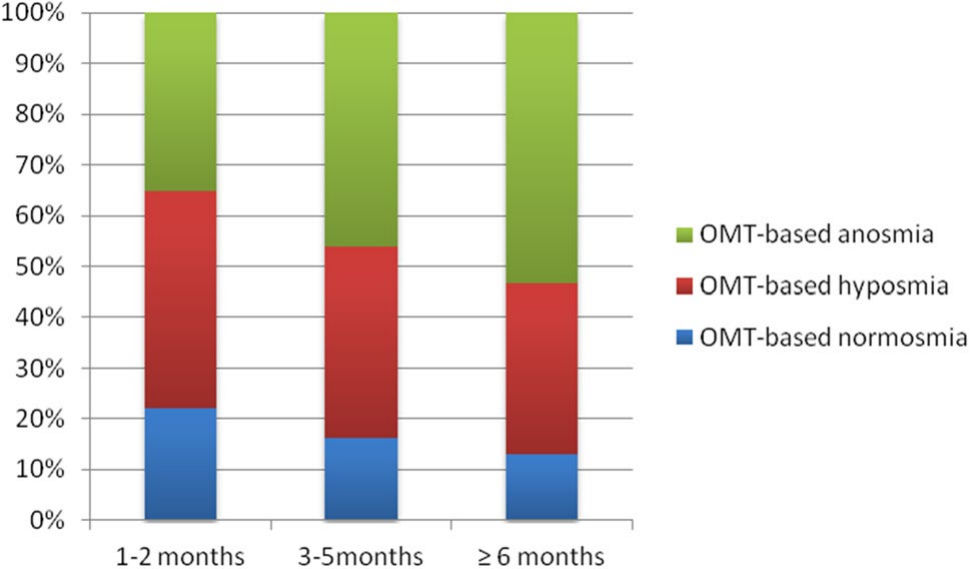
Distribution of the rates of OMT-based normosmia, OMT-based hyposmia and OMT-based anosmia in groups according to OD duration at the time of testing.

We were also interested in whether the duration of olfactory dysfunction was dependent on its severity. The most severe OMT-based olfactory dysfunction, anosmia monotonically increased as the duration of olfactory dysfunction increased from 1–2 months through 3–5 months to more than 6 months, while OMT-based normosmia decreased correspondingly (Fig. 5). Notably, the ratios of the participants with durations of more than 6 months, 3–5 months, and 1–2 months were 16.1%, 65.1%, and 18.9%, respectively. These results indicate that some, but not all, severe olfactory dysfunctions tend to persist beyond three or six months as a post-COVID-19 symptom. The distribution of the rates of normosmia, hyposmia, and anosmia in groups of participants according to the duration of OD is shown in Fig. 5.

### Participants with loss of smell as the only symptom of SARS-CoV-2 infection

According the questionnaire, 24.8% (204/824) of all enrolled participants reported loss of smell as the only symptom of SARS-CoV-2 infection (i.e. no other common symptoms of COVID-19). Loss of smell as the only symptom of COVID-19 was observed in 24.1% (144/598) of women and 26.6% (60/226) of men.

In this regard, a noticeable association emerged between the severity of OD confirmed by the OMT and the reported incidence of olfactory loss as the only symptom of COVID-19, suggesting that loss of smell alone was associated with more severe smell impairment. The majority of participants who reported loss of smell as the only symptom of COVID-19 had OMT-confirmed anosmia (45.6% = 93/204) or hyposmia (38.2% = 78/204).

### Correlation between BMI and olfactory dysfunction

**Figure 6:**
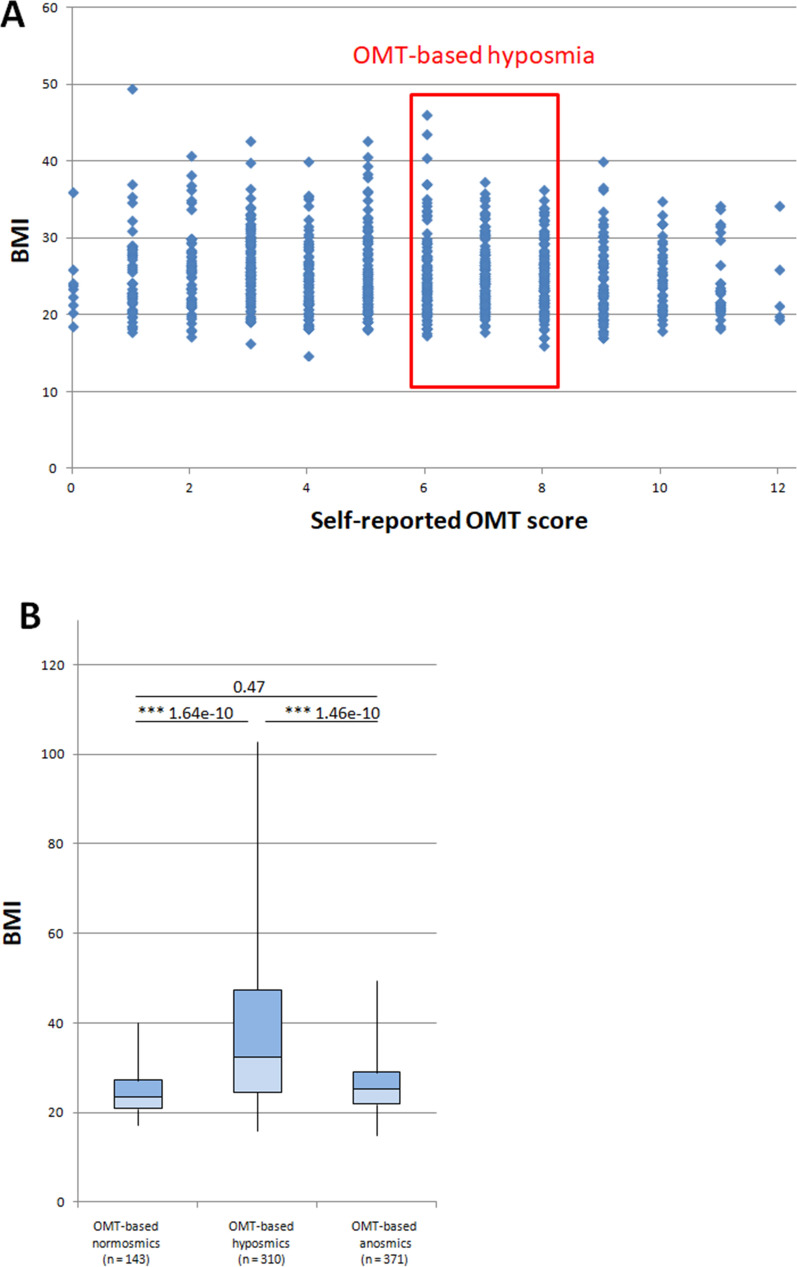
Correlation between BMI and olfactory dysfunction (**A**) Scatter plot of OMT scores vs. BMI (**B**) Statistical distribution of BMI for OMT-based normosmic participants**,** OMT-based hyposmic participants and OMT-based anosmic participants. The Tukey‒Kramer test revealed significant differences in BMI between OMT-based normosmic and OMT-based hyposmic participants as well as between OMT-based hyposmic and OMT-based anosmic participant (***P < 0.001).

We also correlated olfactory dysfunction with BMI, calculated on the basis of self-reported height and weight. According to the BMI, 16.1% (23/143), 18.1% (56/310) and 18.6% (69/371) of participants were obese (BMI ≥ 30) in the OMT-based normosmia, OMT-based hyposmia and OMT-based anosmia groups, respectively. On average 18.0% (148/824) of all participants were obese (Fig. 6A).

Statistical analyses (Tukey‒Kramer test) revealed significant differences in BMI between OMT-based normosmic participants vs. OMT-based hyposmic participants and OMT-based hyposmic participants vs. OMT-based anosmic participants (Fig. 6B). The highest BMI median was found in the group of OMT-based hyposmic participants.

We found a negative correlation between BMI and OMT score. According to the Spearman correlation, participants with higher BMI scores had lower OMT scores (Spearman correlation, BMI vs. OMT score, r value = − 0.067). This suggests that OD was more severe in obese participants.

### Qualitative olfactory dysfunction: self-report of parosmia and phantosmia

A total of 43.2% (356/824) of participants self-reported a distorted olfactory perception (parosmia) and 36.9% (304/824) of participants stated that they had olfactory perception even without the presence of an odorous substance (phantosmia). Sex differences were relatively high for both types of qualitative olfactory dysfunction (OD). Of the total number of women 598, 275 (46.0%) rated their olfactory disturbance as parosmia. Parosmia was less common in men: of the total number of men, 226, 81 (35.8%) indicated that they perceived odors in a different way than normal. Phantosmia was reported by 36.9% (304/824) of participants, and its incidence was much higher among women, 245 of 598 (41.0%) than among men, 59 of 226 (26.1%).

**Figure 7:**
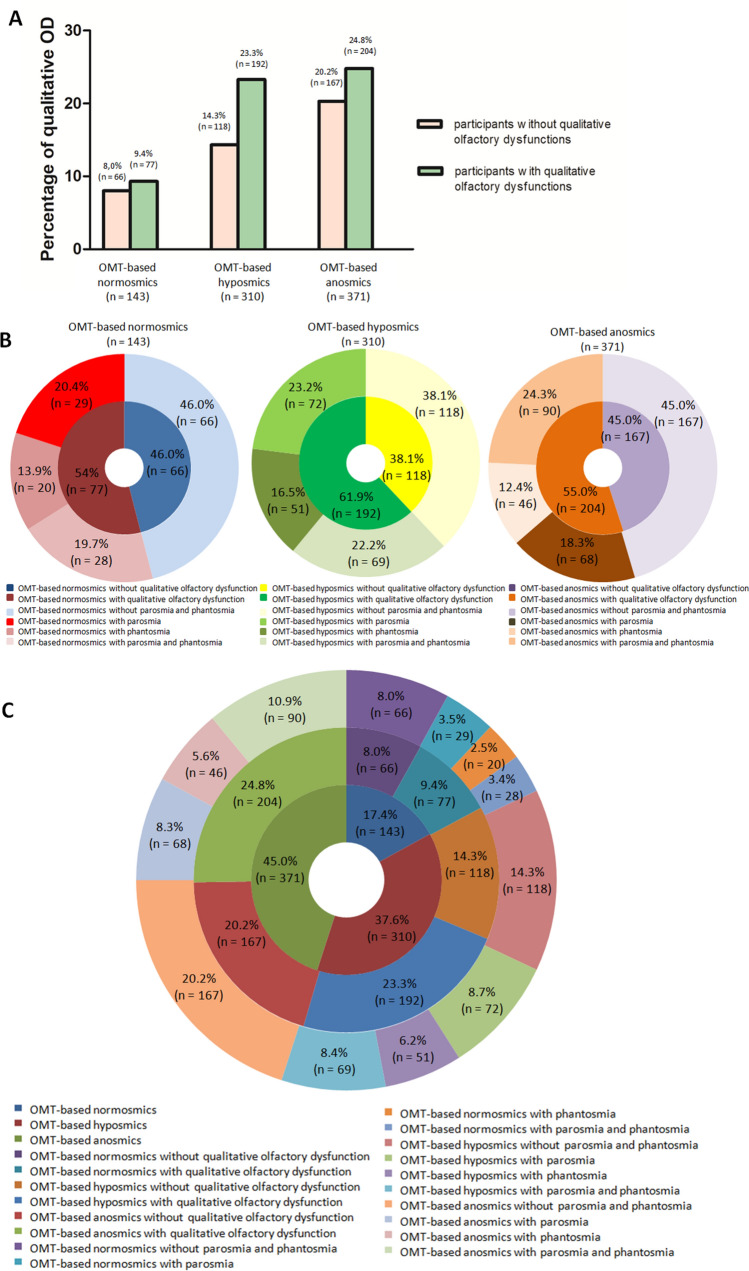
Percentage of qualitative OD (parosmia, phantosmia). (**A**) Percentage of participants with or without qualitative ODs in individual OMT-based groups. (**B**) Percentage of qualitative ODs among OMT-based normosmic, OMT-based hyposmic and OMT-based anosmic participants. (**C**) Concurrent occurrence of quantitative (OMT-based hyposmia, OMT-based anosmia) and qualitative (parosmia, phantosmia) olfactory dysfunctions. Qualitative dysfunctions are also shown for OMT-based normosmia.

Based on participant self-reports, we also investigated the occurrence of qualitative OD, parosmia or phantosmia or their concurrent occurrence in groups of OMT-based normosmic, OMT-based hyposmic and OMT-based anosmic participants. The ratio of participants with qualitative ODs to participants without qualitative ODs was the highest in the group of OMT-based hyposmic participants (Fig. 7A). One qualitative OD or both ODs together were found in 54.0%, 61.9% and 55.0% of OMT-based normosmic, hyposmic and anosmic participants, respectively (Fig. 7B). The results are summarized in Fig. 7C and Supplementary Table 2.

Self-report of qualitative olfactory dysfunctions showed that parosmia occurred in 20.4% of OMT-based normosmic, 23.2% of OMT-based hyposmic and 18.3% of OMT-based anosmic participants. Phantosmia was self-reported by 13.9% of OMT-based normosmic, 16.5% of OMT-based hyposmic and 12.4% of OMT-based anosmic participants. The concurrent occurrence of parosmia and phantosmia was reported by 19.7% of OMT-based normosmic, 22.2% of OMT-based hyposmic and 24.3% of OMT-based anosmic participants (Fig. 7B).

A total of 46.0% of OMT-based normosmic, 38.1% of OMT-based hyposmic and 45.0% of OMT-based anosmic participants did not report any qualitative OD (Fig. 7B).

### Self-report of taste dysfunction

**Figure 8:**
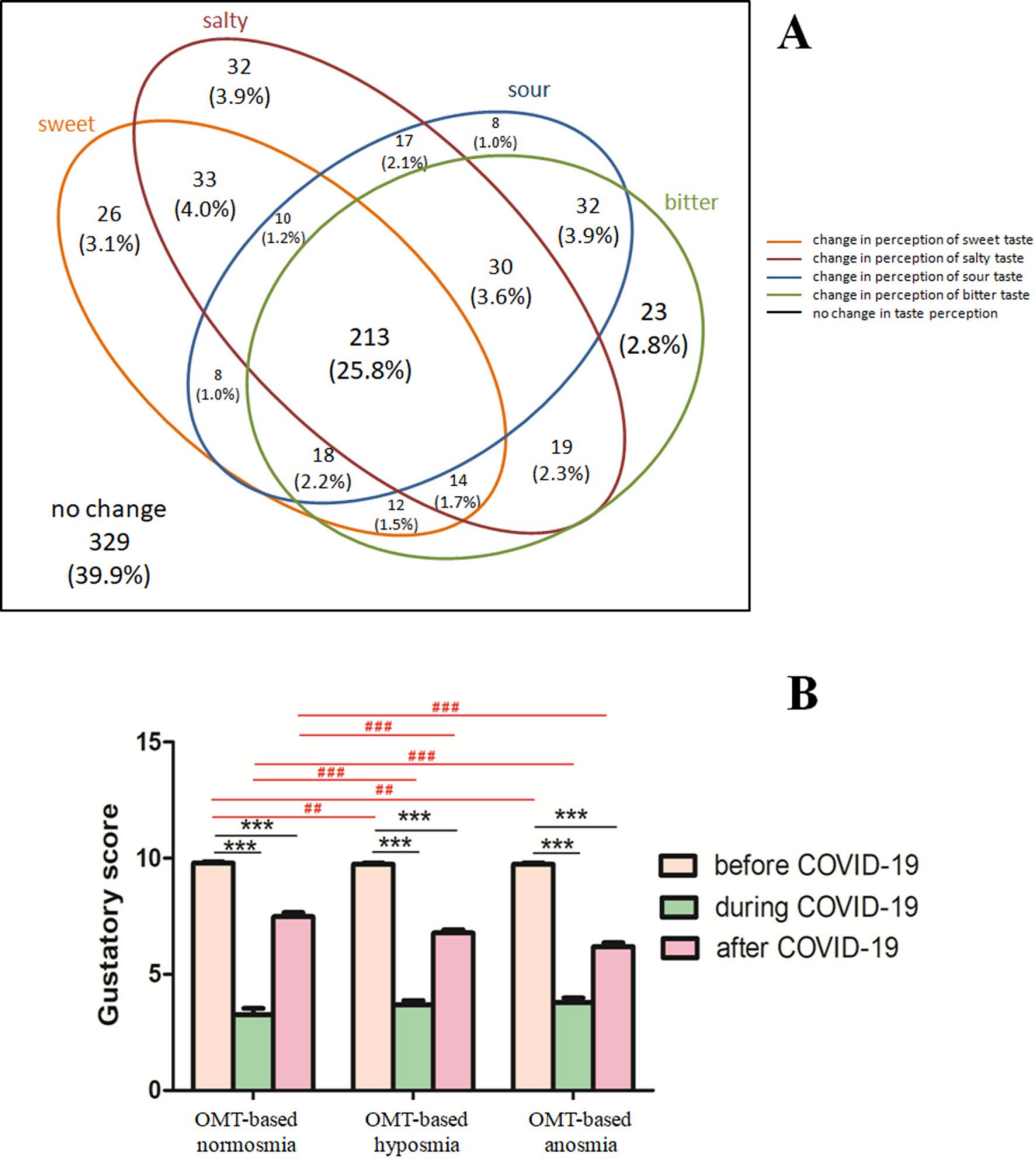
Altered taste perception after COVID-19 infection. (**A**) Venn diagram illustrating the numbers and frequencies of participants reporting changes in taste perception. Overlaps between affected tastes can be seen (**B**) Gustatory self-report results. The graph shows the average gustatory scores for each type of OMT-based group at the three observation times. Data are shown as the means ± SEMs. Statistical test: Two-way ANOVA test (***P value <  0.001); post hoc test: Bonferroni's post hoc test (^##^P value < 0.01; ^###^P value < 0.001).

As smell and taste are closely linked, the loss of smell can affect taste detection. In our study, based on self-report, 89.0% (733/824) of participants rated their taste on a numeric scale (1 = no perception and 10 = perfect perception in case of smell and taste) by a value < 10 during infection. We also asked participants which taste quality had changed since the onset of infection. Most of them experienced a change in more than one taste quality (Fig. 8A). Notably, the changes concerned the individual tastes quite equally (Supplementary Table 3). The frequencies of changes in individual tastes are summarized in Fig. 8A.

According to self-report of taste on the numeric scale (from 1 = no perception to 10 = perfect perception) before, during and after COVID-19, 12.9% of all participants reported that their taste improved to the level before COVID-19 infection (they rated their taste with the same numbers before and after COVID-19, while during the disease with lower number, e.g. 10, 8, 10, or 9, 7, 9). We found significant differences in gustatory self-report rates before vs. during COVID-19 and before vs. after COVID-19 in all OMT-based groups (Fig. 8B; P value < 0.001). Based on self-reports, significant differences were found in the improvement of taste dysfunction (gustatory scores) after COVID-19 between groups of OMT-based normosmic vs. OMT-based hyposmic participants and OMT-based normosmic vs. OMT-based anosmic participants (Fig. 8B; P value < 0.001).

Associations between olfactory and gustatory dysfunctions during COVID-19 were also investigated. Based on self-reports of smell and taste, 10.4% (86/824) of participants reported solely olfactory disturbances without loss of taste. However, most of the participants (88.4%, 728/824) complained of both smell and taste disturbances.

## Discussion

In most COVID-19 patients, loss of smell is usually transient and recovers spontaneously within days or weeks^7,35^. However, in some patients (approximately 10%), olfactory dysfunction persists for a longer period of time^25,26^. Although the loss of smell does not directly endanger a person’s life, it can severely impair the quality of life and can cause other health problems. Regrettably, in Slovakia, there is no examination of smell by validated olfactory tests in clinical practice that would enable the evaluation of OD. Our study was conducted in collaboration with otolaryngologists, who set up a network of outpatient clinics throughout the country, where patients with persistent OD can receive medical care. Our initiative has met with great interest. For illustration only, during the first two days after the activation of the website, more than 500 volunteers expressed their interest in smell testing.

### Participants

In this survey, we evaluated a unique group of 824 volunteers with persistent olfactory dysfunction of varying duration and severity after COVID-19 who were motivated to seek treatment from otolaryngologists. The majority of participants took advantage of the follow-up medical examination, and 117 patients benefitted from olfactory training following the instructions of ENT doctors^36^.

Regarding the age composition of the examined participants, we recorded an average age of 39 years, but people over the age of 60 were no exception. This is in accordance with findings showing that OD occurs more often in younger or middle-aged people^37^. The average ages of women and men were similar. However, there was a remarkable sex difference in the number of participants; women comprised 72.5% of the total population of participants. This is consistent with other findings^7,38,39^ showing that women are more frequently affected by olfactory and taste dysfunction due to COVID-19 than men. This predominance may also reflect the greater interest of women in health care.

The occurrence of OD appeared to depend on the SARS-CoV-2 variant^18,29,40,41^. Based on the data provided by the participants, olfactory dysfunction occurred most frequently between November 2020 and February 2021, with a peak in December 2020. During this period, the Alpha variant (B.1.1.7) of SARS-CoV-2 predominated in Central Europe and in Slovakia^42,43^, and this variant has been related to the risk of OD with the highest odds ratio compared to other variants^20,40^. This could explain the higher proportion of participants with OD onset during this time.

Regarding the duration of olfactory impairment, most participants (65.1%) had OD lasting from 3 to 5 months. Importantly, the severity of OD was increased in a group of patients who had suffered from OD for a longer time. We recorded the highest prevalence of hyposmia and anosmia among participants with OD lasting 6 months or more. Contrary to our study, which included only participants who had persistent OD (lasting more than one month), there was an opposite tendency in studies that included all COVID-19 patients with OD, regardless of the duration of olfactory loss^44–46^. In these studies, a spontaneous recovery of smell occurred, and over time, normosmia increased and anosmia decreased.

### Olfactory dysfunction

The main benefit of our initiative was that in a short time and in pandemic conditions, we organized and promoted a simple but reliable method of remote psychophysical smell testing among post-COVID-19 patients who complained of persistent olfactory dysfunction. For this purpose, the reliable and valid Odorized Markers Test^47–49^ was used. The OMT is used in clinical practice in the Czech Republic^47^. The test was correlated with the Sniffin’ Sticks test, and a significant mutual correlation was demonstrated between them (r = 0.5 on p < 0.05)^50^. The OMT results obtained by the participants in our study served as an objective indicator for ENT doctors, on the basis of which they could proceed further.

Using the OMT, impaired smell ability was detected in the vast majority of participants who contracted COVID-19. Persistent hyposmia or anosmia (lasting more than one month) was confirmed by the OMT in 82.6% of respondents. Olfactory testing revealed a predominance of OMT-based anosmia (45.0%) among the participants with OD. This can be explained by the nature of our cohort in which the volunteers applied based on a subjectively perceived smell dysfunction. The majority of OMT-based anosmic participants were women, which is similar to other published studies^37,51^. Why women can develop anosmia to a greater extent than men is not yet known. Similarly, it has not been sufficiently explained why women are at higher risk of developing post-COVID-19 symptoms.

The reliability of olfactory self-report was verified in participants of all OMT-based groups. Although there was a correlation between self-reports and OMT scores, these outcomes did not correspond in all cases. The discrepancy between the results of objective testing and self‐reported impairment of smell was more pronounced in normosmic participants, who tended to underestimate their sense of smell. It could be partially affected by qualitative changes in smell, such as parosmia and phantosmia, which have been complained about by more than 50% of normosmic participants. However, our results, similar to the results of other authors^34,52^ showing that most individuals inaccurately self-report the degree of the olfactory disorder, strongly indicate the need for testing, at least with psychophysical tests.

The rate of OD among patients with COVID-19, according to the current literature, shows high variability^38,53^. These discrepancies could be explained by the differences in the composition of the studied groups of patients (e.g., inpatients in the acute phase of COVID-19 or post-COVID-19 patients) as well as the different methods used (questionnaires, different types of olfactory tests or examinations).

Regarding the possible effect of nasal patency on olfactory dysfunction, statistical analysis showed that self-reported nasal patency decreased significantly during COVID-19 in all OMT-based groups compared to nasal patency before and after COVID-19. However, 20% of participants reported that decreased olfaction was without concurrent reduced nasal patency during COVID-19. Moreover, according to our statistical analysis, OMT-based anosmic participants reported better nasal patency than normosmic participants during COVID-19. These results indicate that the subjective assessment of nasal patency does not correlate with changes in olfaction. This is in agreement with the known fact that OD induced by COVID-19 may occur without reduced nasal patency, such as nasal obstruction caused by swelling of the nasal mucosa experienced during the common cold^54–57^.

In our study, we also analyzed the possible relationship between OD and obesity. The starting point of these correlation analyses was the findings suggesting that obese individuals could have altered smell ability^58,59^. Indeed, we found a negative correlation between BMI and OMT score. Statistical analyses showed significantly higher BMI in hyposmic participants than in normosmic participants. These findings suggest that people with obesity may be at higher risk for olfactory dysfunction.

In addition to quantitative OD, many participants also reported qualitative OD—parosmia and/or phantosmia. The occurrence of both qualitative smell symptoms was higher in women than in men. In contrast to our observations of a higher incidence of parosmia in women, Lerner et al.^60^ found no significant sex differences among respondents with parosmia associated with COVID-19. This discrepancy may be due to the nature of the cohorts. Subjects in the latter study were recruited from otolaryngology and primary care, and parosmia was monitored in a much smaller number of respondents. One or both qualitative ODs were found in more than 50% of participants in all OMT-based groups. Notably, 54% of OMT-based normosmic participants complained of parosmia and/or phantosmia. Parosmia was more common in OMT-based hyposmic participants, and interestingly, concurrent occurrence of both dysfunctions (parosmia and phantosmia) was most frequently reported by OMT-based anosmic patients. In general, it is accepted that parosmia may reflect neuronal regeneration during recovery of the olfactory system^59^. In the case of COVID-19, it has also been shown that patients suffer from parosmia mainly in the early phases of recovery (2–3 months from the onset of COVID-19) from quantitative OD^61^.

### Gustatory dysfunction

During the pandemic, several studies showed that not only olfactory but also gustatory dysfunctions can be associated with COVID-19^54,62^. Loss of taste as an early symptom of COVID-19 has attracted less attention than olfactory dysfunction. COVID-19-derived taste dysfunctions remain poorly explained, particularly regarding its duration and the proportion of patients with persistent taste dysfunction^22^.

Regarding the data obtained thus far, the rates of gustatory dysfunctions are in a wide range^54^. In our study, 89.0% of the participants had impaired gustatory function during COVID-19 according to the outcomes of the self-report of taste. Moreover, most of the participants reported changes in more than one taste quality. The observed high prevalence can be explained by the fact that subjective perception of taste is often overestimated. Another explanation could be that patients report disorders of smell and taste interchangeably^7^. In fact, a small percentage of participants reported loss of smell as their only symptom of COVID-19 and claimed to have impaired taste in another part of the questionnaire. Some of them probably did not distinguish between smell and taste or perceived it together.

Although SARS-CoV-2 can infect cells of both olfactory and gustatory systems^63,64^, altered taste can be attributed to impaired retronasal olfaction^7^. Moreover, injury to taste receptor cells can occur indirectly by inflammatory cytokines^65^. This may also explain the low percentage of participants in our study who reported a recovery of taste after COVID-19. Most likely, some of them still had olfactory dysfunction rather than taste impairment. A rare study using objective taste testing clearly showed that the percentage of patients with gustatory dysfunction was much lower than that found in the self-report of taste^66^. This suggests the need to use objective testing even in the case of taste. Based on self-reports, we found significant differences in the improvement of taste dysfunction after COVID-19, with better rates for OMT-based normosmic and hyposmic participants and worse rates for OMT-based anosmic participants. This is consistent with findings^28^ that the severity of chemosensory dysfunction at onset negatively correlates with recovery.

Although the present study represented a cross-section of the Slovak population affected by persistent OD during the second wave of the COVID-19 pandemic, it has some limitations. First, there was a high prevalence of long-lasting olfactory dysfunction among participants. The composition of the cohort was influenced by the fact that patients with longer persistence of olfactory dysfunction were more likely to participate in this study and were also more motivated to seek treatment from otolaryngologists. Determination of parosmia and phantosmia was based on the subjective evaluation of the participants, and gustatory function was also not measured psychophysically*.* The absence of follow-up of the participants is another limitation; therefore, we could not determine the actual recovery rate of OD in patients. Finally, using the Odorized Markers Test with only six odors (even though the test was previously validated and proven to be a reliable method of detecting ODs) may have caused diagnostic errors in differentiation among normosmia, hyposmia and anosmia. Although a more complex olfactory test would have a higher diagnostic value, the OMT was the first choice for this study because it allowed remote psychophysical self-testing of smell during pandemic restrictions.

## Conclusion

The growing number of patients with loss of smell after COVID-19 prompted the creation of the project "Smell and COVID-19". The results of remotely performed smell testing using the validated OMT during the second wave of the pandemic in Slovakia showed a high prevalence of persistent OD lasting from one month to 1 year at the time of testing. After our evaluation of the questionnaire, patients were given a recommendation for professional medical care. The results of psychophysical olfactory testing served as an objective indicator for ENT doctors.

In addition, the persistent loss of sense of smell due to COVID-19 has drawn the attention of professionals to the importance of this sense, which has only rarely been addressed in clinical practice in Slovakia thus far.

Finally, given the large number of COVID-19 cases, there are probably several million people worldwide with long-term olfactory problems. Thus the problem of loss of smell will not disappear anytime soon.

## Material and methods

### Participants

This cross-sectional study was conducted in Slovakia during the pandemic, during the period between March 2021 and June 2021.

The study was approved by the Ethics Committee of the Košice self-governing region, Slovakia. All participants provided informed consent to participate in this study. All methods were performed in accordance with the relevant guidelines and regulations. The inclusion criteria of this study were as follows: post-COVID-19 patients with persistent olfactory dysfunction (OD) lasting 1 month or more. The exclusion criteria of this study were as follows: participants who did not consent to participate in this study, registered participants without a questionnaire or with an incomplete questionnaire, patients with OD lasting less than 1 month and patients with OD for reasons other than COVID-19 infection. A total of 1025 participants showed interest in participating in olfactory testing. Of these, 824 participants with indicated OD due to COVID-19 infection met the criteria for further investigation. Other registrants were excluded from this study.

### Olfactory testing

Due to the pandemic situation, remote smell testing was proposed for post-COVID-19 patients with persistent OD. For this purpose, the website www.cuch.sk ("cuch" is the Slovak term for "smell") was created and popularized in the mass media. The website contained an online application for olfactory testing, self-testing instructions, an online questionnaire that included olfactory test questions, a description of olfactory training to improve sense of smell, and up-to-date information on olfactory loss in connection with COVID-19.

Based on the online request (by submitting an online application for olfactory testing), the Odorized Markers Test (OMT, manufactured by Centropen, ART. 2589/6) was mailed to the interested participants.

The OMT is a two-part psychophysical screening test designed to test the ability to identify odorous substances, validated by Vodička et al.^47^, and its reliability was further proven^48^. The odorized marker screening test consists of six markers with different synthetic scents of fruits and spices (liquorice, lemon, cinnamon, blueberry, apple, and strawberry). In the first part of the OMT, the subjects were asked to smell the individual markers one after another and spontaneously name each of six odors. Only named odors were scored (even if the odors were not properly named), and points were not awarded for different odors with the same name. The maximum score in this part was six. The second part of the OMT is based on the principle of forced choice. The subjects were asked to smell the markers again and to choose the correct name of the odor from four offered options. Additionally in this part, the maximum score was six. The test can distinguish among normosmia, hyposmia and anosmia^49^. The minimum and maximum possible scores are 0 and 12 points, respectively. An OMT score from 9 to 12 points indicates a normal value, the score from 6 to 8 points indicates hyposmia and a score from 0 to 5 points indicates anosmia.

The participants self-administered the test and recorded their answers in the online questionnaire on our website. After evaluation of the questionnaire, the results of the test were sent to the patients by an e-mail along with a list of participating otolaryngologists they could contact throughout Slovakia. The list of ENT doctors was compiled to cover most of the territory of Slovakia so that participants could visit doctors close to their place of residence. A recommendation to visit some of those doctors was sent to each participant regardless of the severity of OD.

### Self-reported smell, taste and nasal patency

Participants rated their sense of smell and taste and nasal patency before, during and after the infection on a numeric scale ranging from 1 to 10. They were asked to indicate their feelings by choosing an appropriate number on the scale (1 = no perception and 10 = perfect perception in the case of smell and taste, in the case of nasal patency, 1 = completely blocked nose and 10 = free breathing through the nose).

In the self-report, participants were asked the following:

1. Whether they smelled odors differently than usual. A positive response indicated the presence of parosmia.
2. Whether they smelled an odor that was not actually there. A positive response indicated the presence of phantosmia.
3. Whether they observed a change in the taste of sweet, salty, sour or bitter.
4. Whether olfactory loss was the only symptom of COVID-19 they experienced.

In addition, the questionnaire contained questions about age, sex, height, and weight. BMI was calculated based on the data provided. In the questionnaire, participants were also asked to indicate the time when they became infected with SARS-CoV-2 and the duration of olfactory dysfunction (in months) at the time of testing.

### Statistical analysis

For data processing, this study was anonymized. Statistical analysis was performed by Excel Visual Basic code using collected data in regular Excel spreadsheets. Averages, percentages and medians were calculated with either regular Excel functions or Visual Basic code, taking into account missing values or answers in the questionnaire data. For data visualization, the Orange data mining software package version 2.7 was used. GraphPad Prism 5.0 (GraphPad Software Inc., USA) was used for statistical analysis of the collected data. All data were analyzed with the one-way ANOVA (posttest: Tukey‒Kramer test), two-way ANOVA (posttest: Bonferroni's post hoc test), the D'Agostino-Pearson omnibus normality test, Spearman rank-order correlation or Student’s t test. The values are expressed as the means ± standard errors of the mean (SEM). Significant changes are labeled as *P ﻿ < 0.05, ***P ﻿ < 0.001, ^##^P < 0.01 and ^###^P < 0.001.

### Ethics approval

The study was approved by the Ethical committee of the Košice self-governing region, Slovakia.

### Consent to participate and consent to publish

Informed consent was obtained from all individual participants included in the study. Additional informed consent was obtained from all individual participants for whom identifying information is included in this article.

### Supplementary Information

Supplementary Figure S1.

Supplementary Tables.

## Data Availability

All data generated or analysed during this study are included in this published article (and its supplementary information files). The datasets generated during and/or analysed during the current study are available from the corresponding author on reasonable request.

## References

[1] Hannum ME (2022). Taste loss as a distinct symptom of COVID-19: A systematic review and meta-analysis. Chem. Senses.

[2] Hajikhani B (2020). Olfactory and gustatory dysfunction in COVID-19 patients: A meta-analysis study. Physiol. Rep..

[3] Butowt R, von Bartheld CS (2021). Anosmia in COVID-19: Underlying mechanisms and assessment of an olfactory route to brain infection. Neuroscientist.

[4] von Bartheld CS, Hagen MM, Butowt R (2020). Prevalence of chemosensory dysfunction in COVID-19 patients: A systematic review and meta-analysis reveals significant ethnic differences. medRxiv.

[5] Iravani B, Arshamian A, Lundström JN (2022). Loss of olfactory sensitivity is an early and reliable marker for COVID-19. Chem. Senses.

[6] Qiu C (2020). Olfactory and gustatory dysfunction as an early identifier of COVID-19 in adults and children: An international multicenter study. Otolaryngol. Head Neck Surg. (United States).

[7] Whitcroft KL, Hummel T (2020). Olfactory dysfunction in COVID-19: Diagnosis and management. JAMA.

[8] Hoffmann M (2020). SARS-CoV-2 cell entry depends on ACE2 and TMPRSS2 and is blocked by a clinically proven protease inhibitor. Cell.

[9] Brann DH (2020). Non-neuronal expression of SARS-CoV-2 entry genes in the olfactory system suggests mechanisms underlying COVID-19-associated anosmia. Sci. Adv..

[10] Baxter BD (2020). Transcriptional profiling reveals potential involvement of microvillous TRPM5-expressing cells in viral infection of the olfactory epithelium. bioRxiv Prepr. Serv. Biol..

[11] Chen M (2020). Elevated ACE2 expression in the olfactory neuroepithelium: Implications for anosmia and upper respiratory SARS-CoV-2 entry and replication. bioRxiv.

[12] Heydel JM (2013). Odorant-binding proteins and xenobiotic metabolizing enzymes: Implications in olfactory perireceptor events. Anat. Rec. (Hoboken).

[13] Villar PS, Delgado R, Vergara C, Reyes JG, Bacigalupo J (2017). Energy requirements of odor transduction in the chemosensory cilia of olfactory sensory neurons rely on oxidative phosphorylation and glycolytic processing of extracellular glucose. J. Neurosci..

[14] Bryche B (2020). Massive transient damage of the olfactory epithelium associated with infection of sustentacular cells by SARS-CoV-2 in golden Syrian hamsters. Brain. Behav. Immun..

[15] Schwob JE (2002). Neural regeneration and the peripheral olfactory system. Anat. Rec..

[16] Shelton JF (2022). The UGT2A1/UGT2A2 locus is associated with COVID-19-related loss of smell or taste. Nat. Genet..

[17] Torabi A (2020). Proinflammatory cytokines in the olfactory mucosa result inCOVID-19 induced anosmia. ACS Chem. Neurosci..

[18] Karamali K, Elliott M, Hopkins C (2022). COVID-19 related olfactory dysfunction. Curr. Opin. Otolaryngol. Head Neck Surg..

[19] Najafloo R (2021). Mechanism of anosmia caused by symptoms of COVID-19 and emerging treatments. ACS Chem. Neurosci..

[20] Stuck BA, Menzel S, Laudien M, Hintschich CA, Hummel T (2023). COVID-19-induced olfactory loss. J. Allergy Clin. Immunol..

[21] Cantuti-Castelvetri L (2020). Neuropilin-1 facilitates SARS-CoV-2 cell entry and infectivity. Science.

[22] Hopkins C, Lechien JR, Saussez S (2021). More that ACE2? NRP1 may play a central role in the underlying pathophysiological mechanism of olfactory dysfunction in COVID-19 and its association with enhanced survival. Med. Hypotheses.

[23] Finlay JB (2022). Persistent post-COVID-19 smell loss is associated with inflammatory infiltration and altered olfactory epithelial gene expression. bioRxiv.

[24] Ohla K (2022). A follow-up on quantitative and qualitative olfactory dysfunction and other symptoms in patients recovering from COVID-19 smell loss. Rhinology.

[25] Boscolo-Rizzo P (2021). Self-reported smell and taste recovery in coronavirus disease 2019 patients: A one-year prospective study. Eur. Arch. Otorhinolaryngol..

[26] Vaira LA (2022). The effects of persistent olfactory and gustatory dysfunctions on quality of life in long-COVID-19 patients. Life (Basel, Switzerland).

[27] Tan, B. K. J. *et al.* Prognosis and persistence of smell and taste dysfunction in patients with covid-19: Meta-analysis with parametric cure modelling of recovery curves. *BMJ.* **378**, (2022). 10.1136/bmj-2021-069503 PMC9326326 35896188

[28] Ferreli F (2022). Long-standing gustatory and olfactory dysfunction in COVID-19 patients: A prospective study. Eur. Arch. Otorhinolaryngol..

[29] Hintschich CA (2022). Persisting olfactory dysfunction in post-COVID-19 is associated with gustatory impairment: Results from chemosensitive testing eight months after the acute infection. PLoS ONE.

[30] Soter A (2008). Accuracy of self-report in detecting taste dysfunction. Laryngoscope.

[31] Nørgaard HJ, Fjaeldstad AW (2021). Differences in correlation between subjective and measured olfactory and gustatory dysfunctions after initial ear, nose and throat evaluation. Int. Arch. Otorhinolaryngol..

[32] Lechien JR (2020). Objective olfactory evaluation of self-reported loss of smell in a case series of 86 COVID-19 patients. Head Neck.

[33] Vaira LA (2020). Olfactory and gustatory function impairment in COVID-19 patients: Italian objective multicenter-study. Head Neck.

[34] Mazzatenta A (2020). Smell and taste in severe CoViD-19: Self-reported vs. testing. Front. Med..

[35] Hopkins C, Surda P, Whitehead E, Kumar BN (2020). Early recovery following new onset anosmia during the COVID-19 pandemic—An observational cohort study. J. Otolaryngol. Head Neck Surg..

[36] Doležal P (2022). Results of treatment of olfactory disorders after COVID-19 disease using olfactory training. Otorinolaryngol. Foniatr..

[37] Topal K, Atalay F, Kars A (2021). Cacosmia and cacogeusia in patients with persistent anosmia and ageusia due to COVID-19. Eur. J. Rhinol. Allergy.

[38] Printza A, Constantinidis J (2020). The role of self-reported smell and taste disorders in suspected COVID-19. Eur. Arch. Otorhinolaryngol..

[39] Ahmed MM (2021). Gender difference in perceived symptoms and laboratory investigationsin suspected and confirmed COVID-19 cases: A retrospective study. J. Prim. Care Community Health.

[40] Whitaker M (2022). Variant-specific symptoms of COVID-19 among 1,542,510 people in England. medRxiv.

[41] Menni C (2022). Symptom prevalence, duration, and risk of hospital admission in individuals infected with SARS-CoV-2 during periods of omicron and delta variant dominance: A prospective observational study from the ZOE COVID Study. Lancet.

[42] Brejová B (2021). Sequencing SARS-CoV-2 in Slovakia: An unofficial genomic surveillance report. medRxiv.

[43] Stadtmüller M (2022). Emergence and spread of a sub-lineage of SARS-CoV-2 Alpha variant B.1.1.7. in Europe, and with further evolution of spike mutation accumulations shared with the Beta and Gamma variants. Virus Evol..

[44] Vaira LA (2020). Smell and taste recovery in coronavirus disease 2019 patients: A 60-day objective and prospective study. J. Laryngol. Otol..

[45] Otte MS, Eckel HNC, Poluschkin L, Klussmann JP, Luers JC (2020). Olfactory dysfunction in patients after recovering from COVID-19. Acta Otolaryngol..

[46] Huart C (2021). Systemic corticosteroids in coronavirus disease 2019 (COVID-19)-related smell dysfunction: An international view. Int. Forum Allergy Rhinol..

[47] Vodička J, Pellant A, Chrobok V (2007). Screening of olfactory function using odourized markers. Rhinology.

[48] Vodička J (2011). Normative data of subjective olfactory tests for the Czech population. Otorhinolaryngol. Phoniatr..

[49] Brothánková P, Vodička J, Pospíchalová K (2022). Test–retest assessment of the olfactory test reliability (Odorized Markers Test). Ces. a Slov. Neurol. a Neurochir..

[50] Pospichalova K, Vodicka J, Kopal A (2016). New test of odor pleasantness in Parkinson’s disease. Funct. Neurol..

[51] Fernández-De-las-peñas C (2022). Female sex is a risk factor associated with long-term post-COVID related-symptoms but not with COVID-19 symptoms: The LONG-COVID-EXP-CM multicenter study. J. Clin. Med..

[52] Doty RL, Smith R, Mckeown DA, Raj J (1994). Tests of human olfactory function: Principal components analysis suggests that most measure a common source of variance. Percept. Psychophys..

[53] Moein ST (2020). Smell dysfunction: A biomarker for COVID-19. Int. Forum Allergy Rhinol..

[54] Lechien JR (2020). Olfactory and gustatory dysfunctions as a clinical presentation of mild-to-moderate forms of the coronavirus disease (COVID-19): A multicenter European study. Eur. Arch. Otorhinolaryngol..

[55] Beltrán-Corbellini (2020). Acute-onset smell and taste disorders in the context of COVID-19: A pilot multicentre polymerase chain reaction based case-control study. Eur. J. Neurol..

[56] Gerkin RC (2021). Recent smell loss is the best predictor of COVID-19 among individuals with recent respiratory symptoms. Chem. Senses.

[57] Parma V (2020). More than smell—COVID-19 is associated with severe impairment of smell, taste, and chemesthesis. Chem. Senses.

[58] Stafford LD, Whittle A (2015). Obese individuals have higher preference and sensitivity to odor of chocolate. Chem. Senses.

[59] Peng M, Coutts D, Wang T, Cakmak YO (2019). Systematic review of olfactory shifts related to obesity. Obes. Rev..

[60] Lerner DK (2022). Clinical features of parosmia associated with COVID-19 infection. Laryngoscope.

[61] Hummel T, Lötsch J (2010). Prognostic factors of olfactory dysfunction. Arch. Otolaryngol. Head. Neck Surg..

[62] Klopfenstein T (2022). Third of patients have gustatory dysfunction 9 months after SARS-CoV-2 infection: The ANOSVID study. Int. J. Infect. Dis..

[63] Xu H (2020). High expression of ACE2 receptor of 2019-nCoV on the epithelial cells of oral mucosa. Int. J. Oral Sci..

[64] Sato T (2020). Expression of ACE2 and TMPRSS2 proteins in the upper and lower aerodigestive tracts of rats. bioRxiv.

[65] Cooper KW (2020). COVID-19 and the chemical senses: Supporting players take center stage. Neuron.

[66] Singer-Cornelius T, Cornelius J, Oberle M, Metternich FU, Brockmeier SJ (2021). Objective gustatory and olfactory dysfunction in COVID-19 patients: A prospective cross-sectional study. Eur. Arch. Otorhinolaryngol..

