# Supplementary figures and images for: Remote psychophysical testing of smell in patients with persistent olfactory dysfunction after COVID-19
Source: Sci Rep. 2023 Aug 28;13:14090. doi: 10.1038/s41598-023-41395-9 (PMC10462624; doi:10.1038/s41598-023-41395-9)

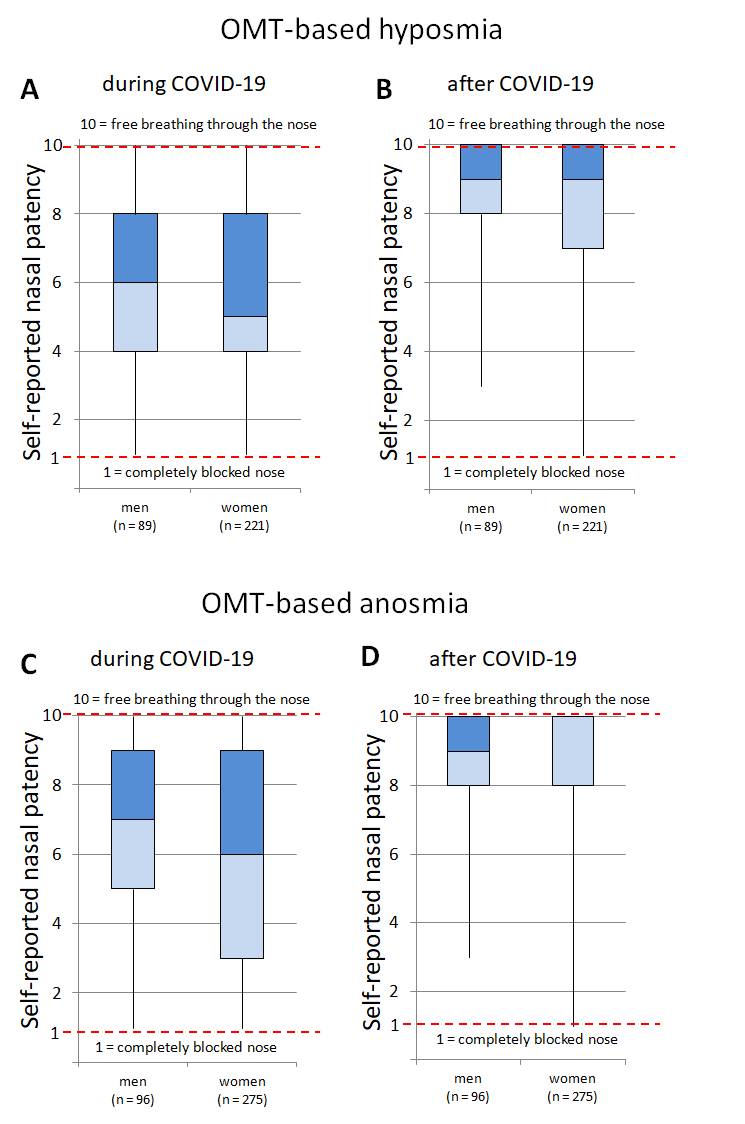

Supplement: Supplementary file 1 — Supplementary Figure S1. [file 41598_2023_41395_MOESM1_ESM.tif]
