# Supplementary material for: Remote psychophysical testing of smell in patients with persistent olfactory dysfunction after COVID-19
Source: Sci Rep. 2023 Aug 28;13:14090. doi: 10.1038/s41598-023-41395-9 (PMC10462624; doi:10.1038/s41598-023-41395-9)
Supplement: Supplementary file 2 — Supplementary Tables. [file 41598_2023_41395_MOESM2_ESM.docx]

| Time of OD onset (mm/yy) | Number of participants | Percentage of all participants |
| --- | --- | --- |
| 01/20 | 4 | 0.5% |
| 02/20 | 1 | 0.1% |
| 03/20 | 1 | 0.1% |
| 04/20 | 1 | 0.1% |
| 05/20 | 2 | 0.2% |
| 06/20 | 1 | 0.1% |
| 07/20 | 2 | 0.2% |
| 08/20 | 1 | 0.1% |
| 09/20 | 20 | 2.4% |
| 10/20 | 81 | 9.9% |
| 11/20 | 136 | 16.6% |
| 12/20 | 248 | 30.2% |
| 01/21 | 173 | 21.1% |
| 02/21 | 89 | 10.9% |
| 03/21 | 44 | 5.4% |
| 04/21 | 13 | 1.6% |
| 05/21 | 1 | 0.1% |
| 06/21 | 2 | 0.2% |

**Supplementary Table 1** The number and percentage of participants according to the time of OD onset

|  | Parosmia | Phantosmia |  |
| --- | --- | --- | --- |
|  | + | - | 20.4% |
| OMT-based normosmia | - | + | 13.9% |
|  | + | + | 19.7% |
|  | + | - | 23.2% |
| OMT-based hyposmia | - | + | 16.5% |
|  | + | + | 22.2% |
|  | + | - | 18.3% |
| OMT-based anosmia | - | + | 12.4% |
|  | + | + | 24.3% |

**Supplementary Table 2** Concurrent occurrence of quantitative (OMT-based hyposmia, OMT-based anosmia) and qualitative (parosmia, phantosmia) olfactory dysfunctions.

| affected taste | sweet | salty | sour | bitter |
| --- | --- | --- | --- | --- |
| % of participants | 40.5 | 44.6 | 40.8 | 43.8 |

**Supplementary Table 3** Altered taste perception during COVID-19 infection


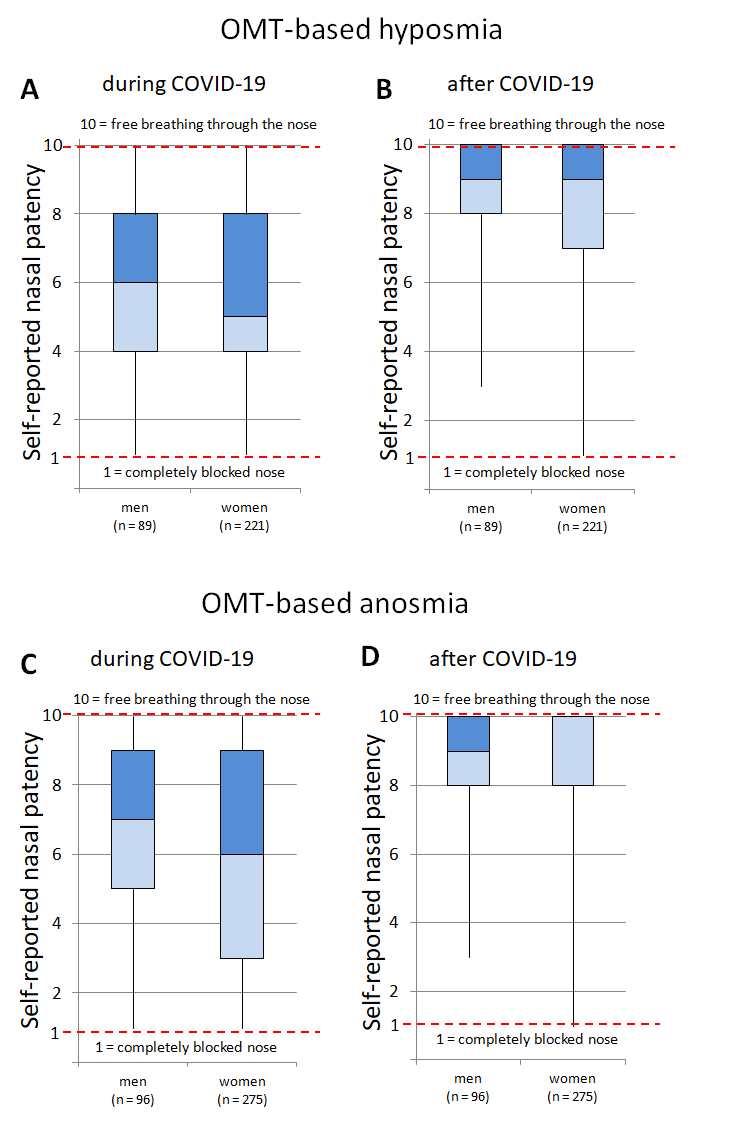


**Supplementary figure 1:** Box plot graphs of self-reported nasal patency for OMT-based hyposmia during (**A**) and after (**B**) COVID-19 and OMT-based anosmia during (**C**) and after (**D**) COVID-19 in men and women. The Tukey‒Kramer test did not reveal significant differences in self-reported nasal patency between men and women.
